# AfroDb: A Select Highly Potent and Diverse Natural Product Library from African Medicinal Plants

**DOI:** 10.1371/journal.pone.0078085

**Published:** 2013-10-30

**Authors:** Fidele Ntie-Kang, Denis Zofou, Smith B. Babiaka, Rolande Meudom, Michael Scharfe, Lydia L. Lifongo, James A. Mbah, Luc Meva’a Mbaze, Wolfgang Sippl, Simon M. N. Efange

**Affiliations:** 1 Chemical and Bioactivity Information Centre, Department of Chemistry, Faculty of Science, University of Buea, Buea, Cameroon; 2 Center Atomic Molecular Physics, Optics and Quantum, Faculty of Science, University of Douala, Douala, Cameroon; 3 Department of Pharmaceutical Sciences, Martin-Luther University of Halle-Wittenberg, Halle (Saale), Germany; 4 Biotechnology Unit, Department of Biochemistry and Molecular Biology, Faculty of Science, University of Buea, Buea, Cameroon; 5 Department of Chemistry, Faculty of Science, University of Buea, Buea, Cameroon; 6 Department of Chemistry, Faculty of Science, University of Douala, Douala, Cameroon; National Cancer Institute at Frederick, United States of America

## Abstract

Computer-aided drug design (CADD) often involves virtual screening (VS) of large compound datasets and the availability of such is vital for drug discovery protocols. We assess the bioactivity and “drug-likeness” of a relatively small but structurally diverse dataset (containing >1,000 compounds) from African medicinal plants, which have been tested and proven a wide range of biological activities. The geographical regions of collection of the medicinal plants cover the entire continent of Africa, based on data from literature sources and information from traditional healers. For each isolated compound, the three dimensional (3D) structure has been used to calculate physico-chemical properties used in the prediction of oral bioavailability on the basis of Lipinski’s “Rule of Five”. A comparative analysis has been carried out with the “drug-like”, “lead-like”, and “fragment-like” subsets, as well as with the Dictionary of Natural Products. A diversity analysis has been carried out in comparison with the ChemBridge diverse database. Furthermore, descriptors related to absorption, distribution, metabolism, excretion and toxicity (ADMET) have been used to predict the pharmacokinetic profile of the compounds within the dataset. Our results prove that drug discovery, beginning with natural products from the African flora, could be highly promising. The 3D structures are available and could be useful for virtual screening and natural product lead generation programs.

## Introduction

Drug design and discovery efforts have often resorted to natural sources for hit/lead compound identification [1]–[5]. This is because nature is an enormous source for structurally diverse chemical scaffolds from which drugs can be isolated and/or synthesized [6]–[8]. Moreover, natural products (NPs) are unique in that they are often rich in stereogenic centres and cover segments of chemical space which are typically not occupied by a majority of synthetic molecules and drugs [9]–[11]. In addition, it can be verified that the African flora has a huge potential and remains an interesting reservoir for new drugs targeting a variety of diseases [12].

Modern drug discovery efforts also incorporate computer-aided approaches like ligand docking, pharmacophore searching, neural networking and binding free energy calculations of potential drugs towards a target receptor. The rationale behind such *in silico* methods has been to simulate the interaction between a potential drug molecule and its receptor or binding site (often the drug target) using 3D computer models [13]–[14]. Hence, the use of computer modeling in drug discovery otherwise known as computer-aided drug design (CADD) requires a compound library containing 3D structures of potential leads, which need to be screened *in silico*, with the view of identifying hit compounds. If this effort is successful, then the identified hits could be confirmed as active compounds using screening assays. Such a procedure considerably cuts down the cost of drug discovery and development [15].

At the moment, efforts towards drug discovery from the African flora have been limited to random screening of extracts and/or bioassay guided fractionation of extracts from medicinal plant materials, based on information obtained from the ethnobotanical uses of the plants [8]. However, these efforts remain below expectations and their applicability as well as their impact, are not felt at the level of local populations. Moreover, most screening efforts are limited to crude extracts and/or *in vitro* screening, with only little work on clinical development of the identified active molecules. The inclusion of *in silico* methods of drug discovery into the scene would likely foster efforts towards lead optimization and facilitates the entry of most interesting compounds into clinical trials. However, this process requires the development of databases of 3D structures of compounds, which have been isolated from medicinal plants in Africa. It should be mentioned that some of the chemical structures are available in published articles in internationally recognized peer-reviewed journals. However, locally published data (in MSc and PhD theses as well as in local or national journals) may not be readily available to the wider scientific community. Moreover, the absence of 3D structures heavily hampers *in silico* virtual screening. In our continuous efforts to build virtual natural product libraries for compounds isolated from the African flora [16]–[18], it has become necessary to identify selected highly potent compounds from the entire continent, generate their 3D structures and make them available for use in virtual screening campaigns.

With the exponential increase in computer power [19], it has become possible today to carry out successful virtual screening on huge databases like the ZINC library (currently 19,607,982 purchasable compounds) [20]–[21] in a matter of weeks [19], [22]. However, such computer power is currently absent in most research laboratories on the African continent, thoughtless of guaranteeing continuous electrical power supplies for long periods of time. This has therefore necessitated the development of relatively small compound library (∼1000 compounds), known to contain NPs from across the continent, which have recorded activities against a wide range of tropical diseases as well as diseases dominant in rich countries (like cancer and hypertension). We therefore present AfroDb, which is available in several file formats, and which could be highly useful in CADD efforts. An evaluation of the potential oral bioavailability has been carried out by Lipinski criteria [23] in comparison with Dictionary of Natural Products (DNP) [24]. Several parameters related to drug metabolism and pharmacokinetics (DMPK) are also computed. A diversity analysis has also been carried out in comparison with the ChemBridge Diverset library [25].

## Results and Discussion

### Origin and Description of Secondary Metabolites

**Figure 1:**
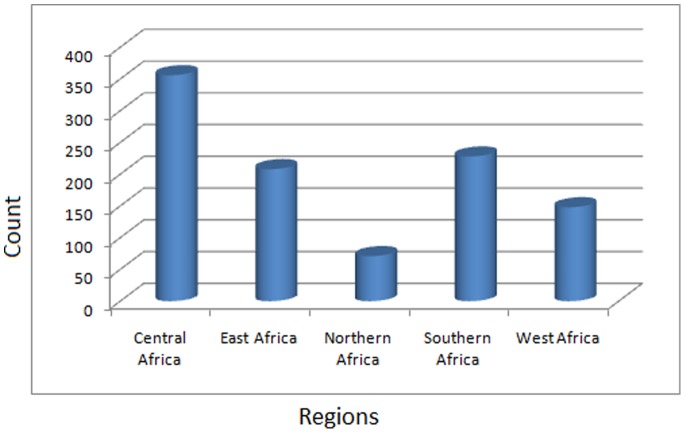
Bar chart showing the distribution of the compounds within AfroDb by region of collection.

The distribution of the compound collection by geographical region of plant origin is shown in Figure 1. This shows that a majority of the compounds with remarkable biological activities were derived from the Central Africa region (35%), followed by Southern Africa (23%) and East Africa (21%). The known biological activities include very specific descriptions (like inhibition or modulation of known drug targets, e.g., prolyl endopeptidase I inhibition, 11β-hydroxysteroid dehydrogenase inhibition, α-glucosidase inhibition, enhancement of cAMP-regulated chloride conductance of cells expressing CFTRΔF508, and snake venom phosphodiesterase I inhibition), while more unspecific classifications include anti-HIV, antisalmonellal, antimalarial, antileishmanial, antitubercular, antitrypanosomal, antitumour, vasodilator, vasorelaxant and hypertensive effects, estrogenic activities, and activity against *Onchocerca gutturosa*.

### Discussion of Lipinski’s Oral Availability Criteria and Property Distribution

**Figure 2:**
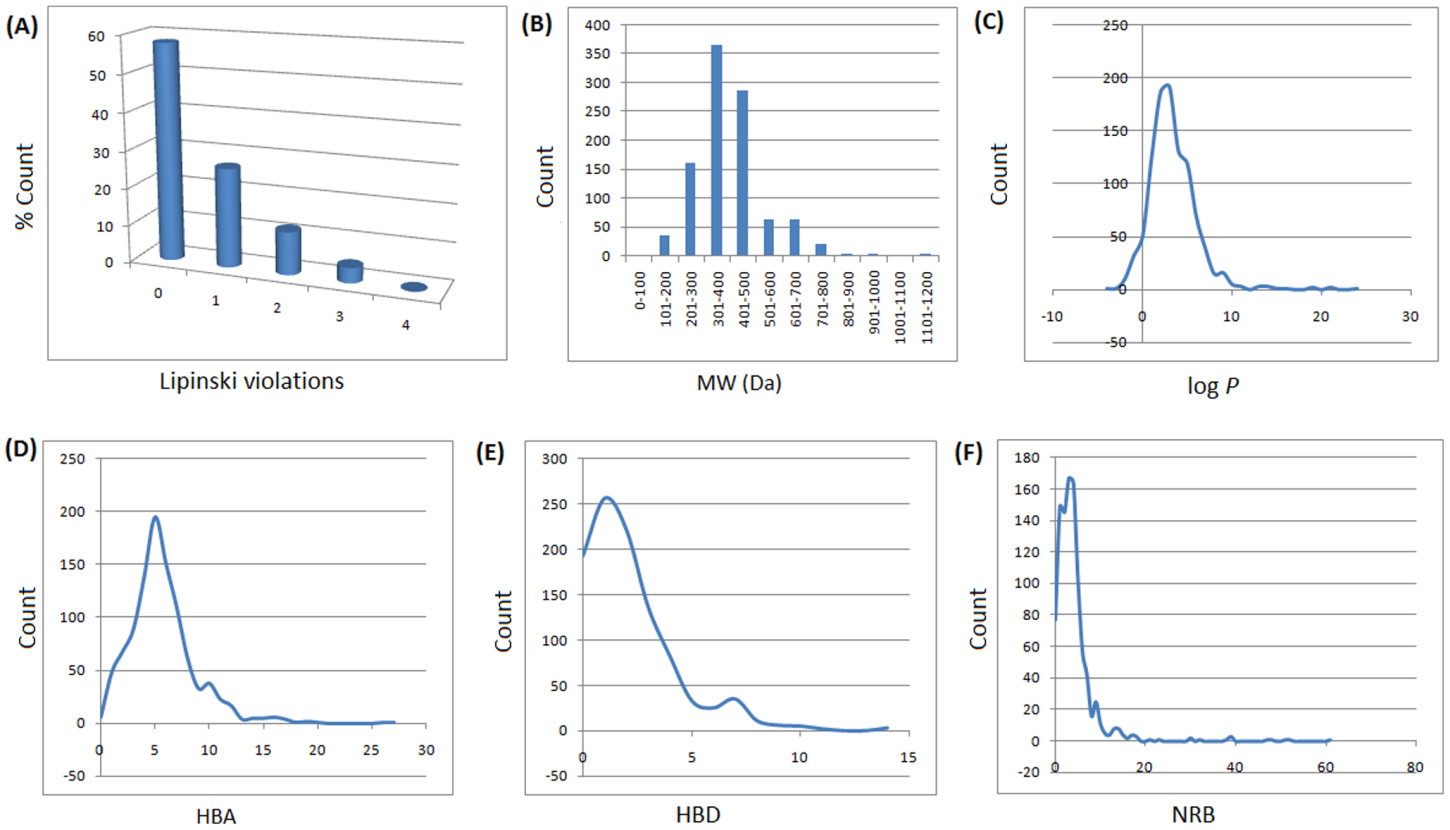
Graph distribution of features that determine “drug-likeness”. (A, B) Histogram of Lipinski violations as a percentage of the AfroDb data set and molar weight distribution, respectively. (C, D, E, F) Distribution curves of the log *P*, HBA, HBD and NRB, respectively for the 1,008 compounds currently in AfroDb. For subfigure B, the *x*-axis label is the lower limit of binned data, e.g. 0 is equivalent to 0 to 100.

**Figure 3:**
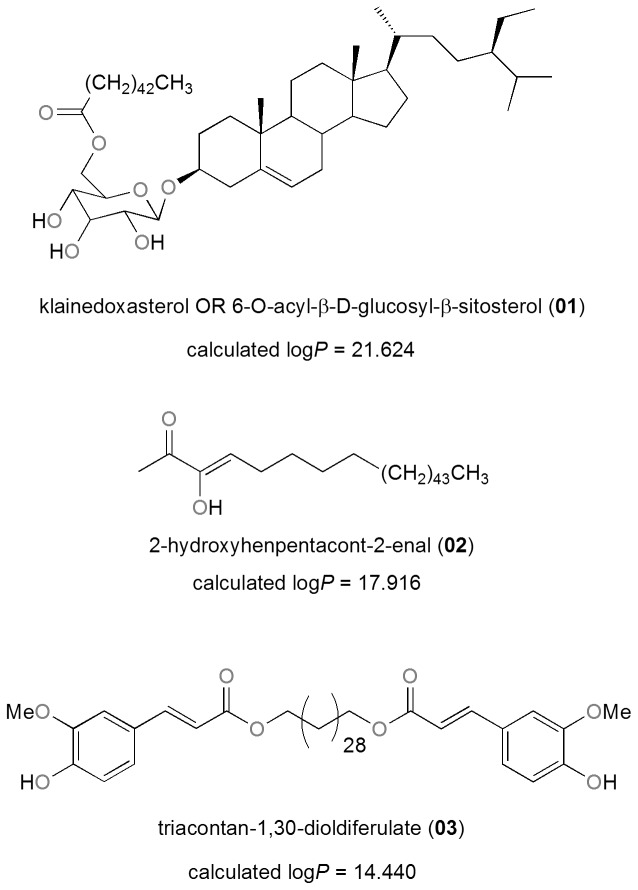
2D structures of the three compounds with log *P* values >14, included in AfroDb.

**Figure 4:**
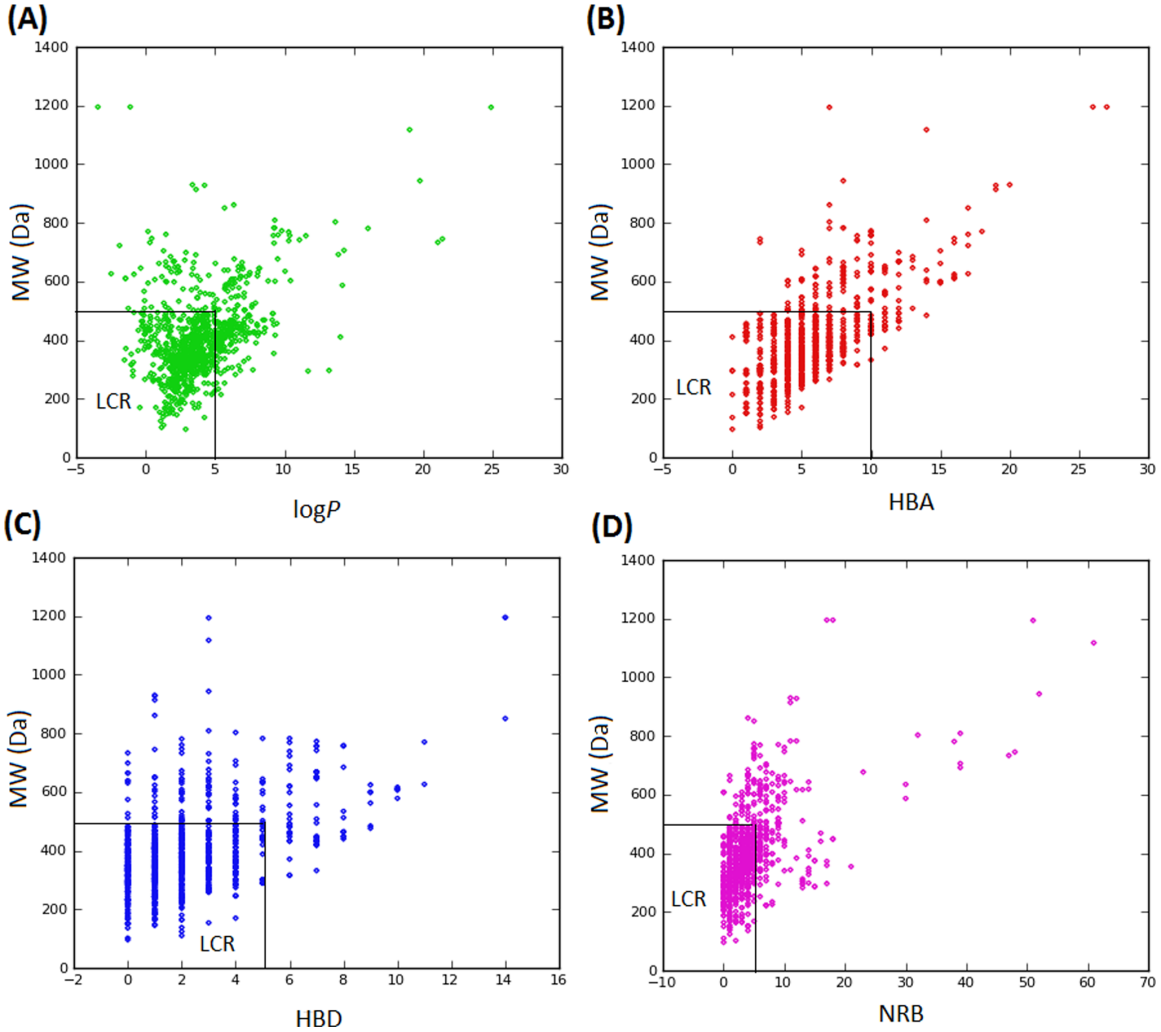
Pairwise comparison of mutual relationships between molecular descriptors. (A) The distribution of the calculated log *P* versus MW, (B) HBA against MW, (C) HBD against MW and (D) NRB versus MW. LCR represents the Lipinski compliant regions.

In modern drug discovery, the identification of lead compounds often involves the development of compound libraries with a high level of molecular diversity within the limits of significant “drug-like” properties [5], [9]. Thus, Lipinski’s criteria [23], generally referred to as the “rule of Five” (ro5), have been used in the evaluation of the likely oral availability of the compounds within the AfroDb database. In summary, Lipinski’s ro5 defines a likely orally available molecule as one for which the molar weight (MW) ≤500 Daltons (Da), the logarithm of the octanol/water partition coefficient representing the lipophilicity factor (log *P*) ≤5, the number of hydrogen bond acceptors (HBA) ≤10 and the number of hydrogen bond donors (HBD) ≤5. A fifth rule dealing with the number of rotatable bonds (NRB) is often added to these, such that NRB ≤5. The distributions of the compound MW, log *P*, HBA and HBD were calculated and used to assess the likely oral availability of AfroDb, as shown in Figure 2. It is noteworthy that natural products exhibit a wide range of flexibility, from rigid conformationally constrained molecules to very flexible compounds. Thus, the number of rotatable bonds (NRB) within the AfroDb library was used as an additional criterion to test for the favourable drug metabolism and pharmacokinetics (DMPK) outcomes. It was observed that 57.8% of the compounds within AfroDb showed no Lipinski violations and 84.3% showed <2 violations (Figure 2A), while the peak of the distribution of the MW lies between 301 and 400 (Figure 2B). The log *P* distribution showed a Gaussian shaped curve with a peak centred at 3.5 log *P* units (Figure 2C), while those of the HBA and HBD rose rapidly to peak values at 5 acceptors and 1 donor respectively. Moreover, both HBA and HBD graphs fell rapidly to maximum values of 27 acceptors and 14 donors (Figures 2D and E). The peak value for NRB was between 3 and 4 (Figure 2F), the graph also falling rapidly to a maximum value of 61 rotatable bonds (RBs). This last parameter indicates the high degree of flexibility of some of the NPs within the database. The MW distribution resembled those previously reported for other “drug-like” NP libraries in the literature [5], [16], [26], with only about 9% of MW >500 Da. In addition, only about 16% of the compounds had log *P* values >5, only about 7% having HBA >10 and only ∼9% had HBD >5. It was observed, however, that some of the compounds had log *P* values as high as >21 units. The exceptionally large calculated logP values (>14) observed in compounds 1 to 3 could be explained by complex partitioning in long chain aliphatic compounds, which could not be properly taken into account by the algorithm employed in the log *P* prediction. The three long chain compounds with log *P*>14 have been shown in Figure 3, with their plant sources and biological activities given in Table 1. The above arguments could be verified by the fact the scatter plots displaying the mutual relationship between the MW versus the other calculated parameters (log *P*, HBA, HBD and NRB), Figure 4, show the highest densities of points within the Lipinski compliance regions (MW <500, −2< log *P*<5, HBA <10, HBD <3), and for which NRB <5.

**Table 1:** Sources and biological activities of metabolites with calculated log *P*>14 found in AfroDb.

Compound	Plant source (country)	Measured activity	Reference
**01**	*Klainedoxa gabonensis* (Cameroon)	xanthine oxidase inhibitory activity	[27]
**02**	*Hugonia castaneifolia* (Tanzania)	antifungal activity against *Cladosporium cucumericum*	[28]
**03**	*Stereospermum acuminatissimum* (Cameroon)	antifungal activities against *Candida* sp.	[29]

### Comparison with the Dictionary of Natural Products (DNP)

**Figure 5:**
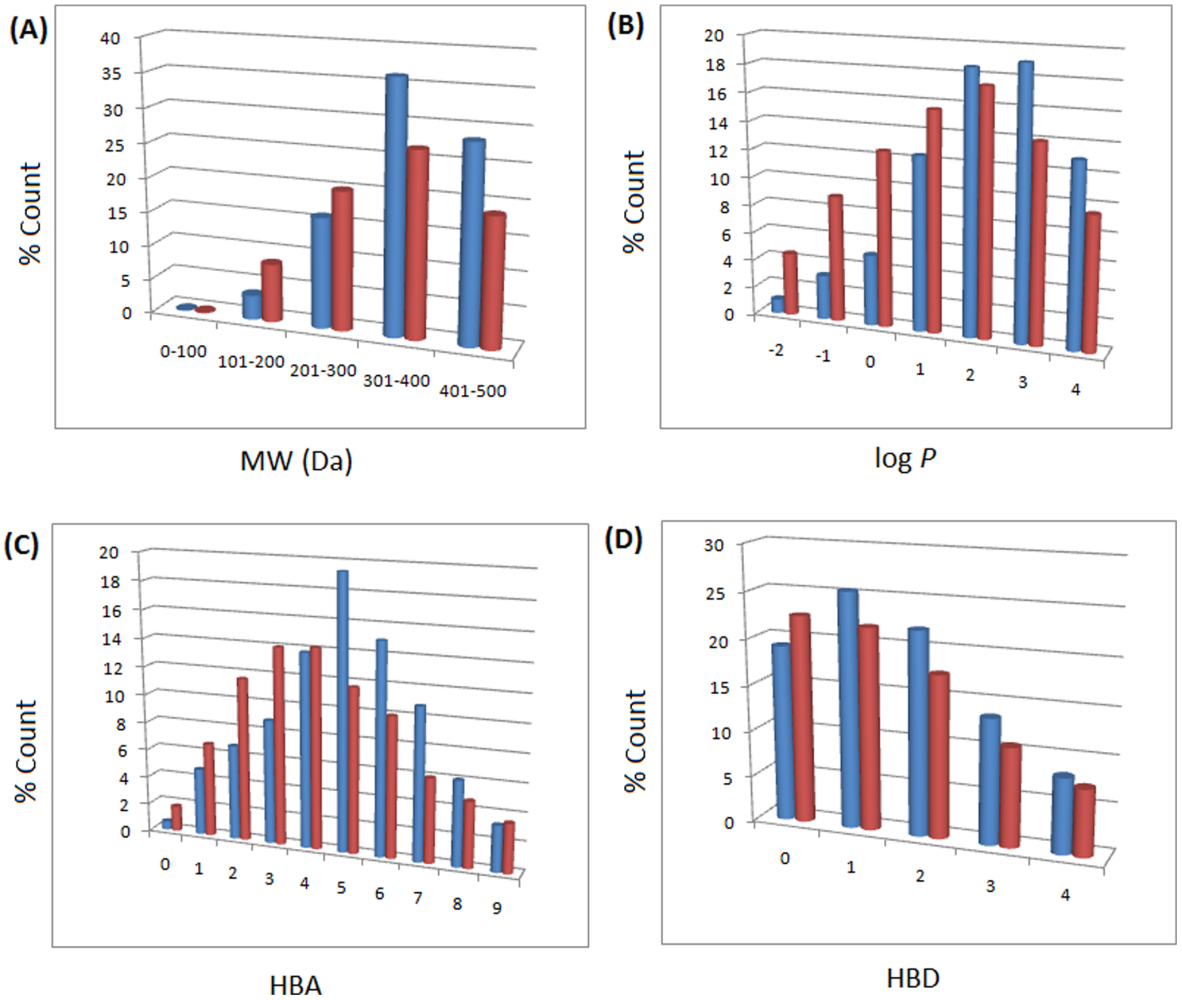
Comparison of property distribution for the two datasets by percentage distributions. (A) MW, (B) log *P*, (C) HBA and (D) HBD. DNP in red and AfroDb in blue. For subfigure B, the *x*-axis label is the lower limit of binned data, e.g. −2 is equivalent to −2 to −1.

The distributions of the individual parameters for AfroDb and the DNP have been compared (Figure 5). These histograms show only data falling within the Lipinski compliance zones (MW <500, −2≤ log *P*<5, HBA <10, and HBD <5), the values being expressed as percentage counts of the respective databases. Our analyses showed an enhancement of the distributions of AfroDb over the DNP for Lipinski properties. As for the MW distribution histograms (Figure 5A), both curves show peaks at 301–400 Da, the AfroDb having >19% enhancement in MW for the region 301 to 500 Da. Below this range, the percentages were reduced for the AfroDb when compared to the DNP.

It is noteworthy that an enhancement of the MW profile is a desirable factor for a more “drug-like library”, according to Lipinski’s criteria. The proportions of the two databases that satisfy Lipinski’s MW property (<500 Da) were about 73% for the DNP, as compared to about 84% for AfroDb. This showed an enhancement of 11.2% for MW values between 301 and 500 Da of AfroDb over the DNP. The maximum values for the log *P* distributions were respectively at 3.5 and 2.5 log *P* units for AfroDb and the DNP (Figure 5B). In addition, a corresponding enhancement of 9.7% for log *P* values between 2 and 5 units was observed for AfroDb over the DNP. As per the HBA and HBD respectively (Figures 5C–D), AfroDb showed improvements of 17.1% for 3< HBA <8 and 10.8% for 0< HBD <4 over the DNP. The peak of the distribution for the HBA for the AfroDb is at 5 acceptors (19.3%) with a significant increase in 7 or 8 acceptors when compared to the DNP (Figure 5C). Similarly, the peak of the distribution for the HBD for the AfroDb is at 1 acceptor (25.4%) with a significant increase in 3 or 4 donors as compared to the DNP (Figure 5D). The overall summary of the four Lipinski parameters for the two datasets thus reveals that the AfroDb library is more “drug-like” than the DNP, indicating that the chances of finding “lead-like” molecules with improved DMPK properties within a library such as AfroDb are quite significant.

### Overall DMPK Compliance of the AfroDb Library

**Figure 6:**
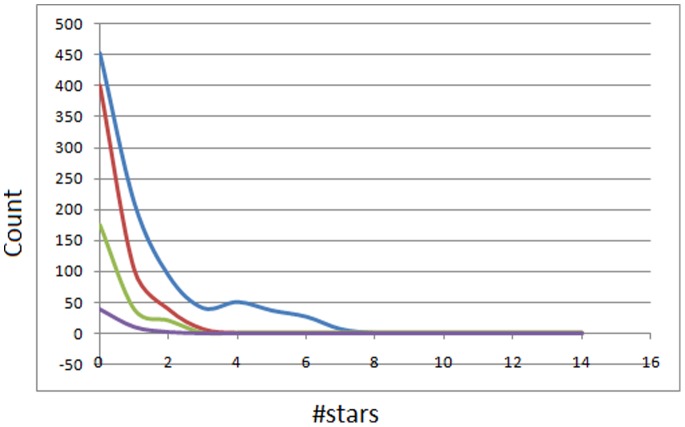
Distribution curves for #stars within the AfroDb library, along with the standard “drug-like”, “lead-like” and “fragment-like” subsets. Blue = AfroDb library, red = “drug-like” subset, green = “lead-like” subset and violet = “fragment-like” subset.

There are 24 molecular descriptors calculated by QikProp software, which are most relevant for the determination the #star parameter [30]. For a given parameter, 1 #star corresponds to the computed property of a molecule falling outside the range for 95% of known drugs. A plot of the #stars parameter (on *x*-axis) against the corresponding counts (on *y*-axis) in the AfroDb is shown within the same set of axes with those of the “drug-like”, “lead-like”, and “fragment-like” standard subsets, (Figure 6). The criteria for the respective standard subsets were defined as (MW <500; log *P*<5; HBD ≤5; HBA ≤10) [23], (150≤ MW ≤350; log *P*≤4; HBD ≤3; HBA ≤6) [31]–[33] and (MW ≤250; −2≤ log *P*≤3; HBD <3; HBA <6; NRB <3) [34]. It was observed that about 48% of the compounds within AfroDb showed #star = 0, while about 79% had #star ≤2. Among the 610 compounds of the “drug-like” subset, 73.4% had pharmacokinetic descriptors within the acceptable range for 95% of known drugs, while 98.9% showed #stars ≤2. The “lead-like” and “fragment-like” subsets were respectively 75.2% and 76.0% compliant for all of the 24 most relevant computed descriptors. The average values for 19 selected computed descriptors for all 4 compound libraries have been shown in Table 2. These values indicate a high probability of finding drug leads within the AfroDb compound library.

**Table 2:** Summary of average predicted pharmacokinetic property distributions of the total AfroDb library in comparison with the various subsets.

Library name	aLib. size	b% compl.	cMW (Da)	dLog*P*	eHBA	fHBD	gNRB
**Total**	1008	48	406	3.99	5.76	1.67	6.30
**Drug-like**	610	73	328	2.99	4.89	1.25	4.24
**Lead-like**	239	75	266	2.44	3.91	0.87	3.43
**Fragment-like**	51	76	219	1.89	3.39	0.60	1.40
**Library name**	h **LogB/B**	i **BIP_caco-2_ (nm** **s^-1^)**	j ***S*** **_mol_ (Å^2^)**	k ***S*** **_mol,hfob_ (Å^2^)**	l ***V*** **_mol_ (Å^3^)**	m **Log** ***S_wat_*** ** (S in** **mol L^-1^)**	n **Log** ***K_HSA_***
**Total**	−0.90	1516	674	415	1265	−5.11	0.59
**Drug-like**	−0.63	1663	568	312	1030	−3.88	0.21
**Lead-like**	−0.57	2032	492	235	860	−3.11	−0.02
**Fragment-like**	−0.29	1983	424	139	712	−2.50	−0.20
**Library name**	o **MDCK**	p **Ind_coh_**	q **Glob**	r **QP_polrz_ (Å^3^)**	s **LogHERG**	t **Log** ***K_p_***	u **# metab**
**Total**	859	0.009	0.84	41.78	−4.68	−2.84	6.13
**Drug-like**	944	0.008	0.87	33.75	−4.33	−2.73	4.85
**Lead-like**	1206	0.005	0.89	27.54	−3.99	−2.55	3.56
**Fragment-like**	1078	0.005	0.91	23.27	−3.90	−2.39	2.28

a Size or number of compounds in library;

b Percentage of compounds with #star = 0;

c Molar weight (range for 95% of drugs: 130–725 Da);

d Logarithm of partitioning coefficient between *n*-octanol and water phases (range for 95% of drugs: −2 to 6);

e Number of hydrogen bonds accepted by the molecule (range for 95% of drugs: 2–20);

f Number of hydrogen bonds donated by the molecule (range for 95% of drugs: 0–6).;

g Number of rotatable bonds (range for 95% of drugs: 0–15);

h Logarithm of predicted blood/brain barrier partition coefficient (range for 95% of drugs: −3.0 to 1.0);

i Predicted apparent Caco-2 cell membrane permeability in Boehringer–Ingelheim scale, in nm/s (range for 95% of drugs: <5 low, >100 high);

j Total solvent-accessible molecular surface, in Å^2^ (probe radius 1.4 Å) (range for 95% of drugs: 300–1000 Å^2^);

k Hydrophobic portion of the solvent-accessible molecular surface, in Å^2^ (probe radius 1.4 Å) (range for 95% of drugs: 0–750 (Å^2^);

l Total volume of molecule enclosed by solvent-accessible molecular surface, in Å^3^ (probe radius 1.4 Å) (range for 95% of drugs: 500–2000 Å^3^);

m Logarithm of aqueous solubility (range for 95% of drugs: −6.0 to 0.5);

n Logarithm of predicted binding constant to human serum albumin (range for 95% of drugs: −1.5 to 1.2);

o Predicted apparent MDCK cell permeability in nm/sec (<25 poor, >500 great);

p Index of cohesion interaction in solids (0.0 to 0.05 for 95% of drugs);

q Globularity descriptor (0.75 to 0.95 for 95% of drugs);

r Predicted polarizability (13.0 to 70.0 for 95% of drugs);

s Predicted IC_50_ value for blockage of HERG K^+^ channels (concern<−5);

t Predicted skin permeability (−8.0 to −1.0 for 95% of drugs);

u Number of likely metabolic reactions (range for 95% of drugs: 1–8).

### Bioavailability Prediction

Two processes determine the bioavailability of a compound; absorption and liver first-pass metabolism [35]. The solubility and permeability of the compound, as well as interactions with transporters and metabolizing enzymes in the gut wall are factors responsible for absorption, while metabolism depends on the functional group types present. The computed parameters used to assess oral absorption revolved around Jorgensen’s famous “Rule of Three” (ro3). According to the ro3, a compliance with all or some of the rules (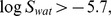
BIP*_caco-2_*>22 nm/s and # primary metabolites <7) is indicative of likelihood to oral availability. Thus, the most important parameters often considered are the predicted aqueous solubility, 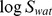, the conformation-independent predicted aqueous solubility, CI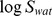, the predicted qualitative human oral absorption, the predicted % human oral absorption. The solubility calculation procedure implemented depends on the similarity property space between the given molecule and its most similar analogue within the experimental training set used to develop the model implemented in QikProp, i.e., if the similarity is <0.9, then the QikProp predicted value is taken. Otherwise, the predicted property, 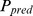
_,_ is adjusted such that:
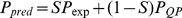
(1)where 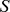 is the similarity, while 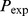
and 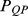
are the respective experimental and QikProp predictions for the most similar molecule within the training set. In equation (1), if 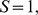
then the predicted property is equal to the measured experimental property of the training set compound. The distribution curves for two of the three determinants for the ro3 (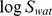
and BIP*_caco-2_*) are shown in Figure S1. In general 43.6% of the AfroDb library was compliant with the ro3, while the respective % compliances for the various subsets were 67.6%, 92.3% and 100% for the “drug-like”, “lead-like” and “fragment-like” libraries. The most remarkable among the individual computed parameters was 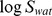, which was met by 73.8% of the compounds within the AfroDb library. This property showed a Gaussian distribution for the “lead-like” and “fragment-like” subsets. The predicted apparent Caco-2 cell permeability, BIP*_caco-2_* (in nm s^−1^), model the permeability of the gut-blood barrier (for non-active transport). Even though the Caco-2 penetration parameter is not often correctly predicted computationally [36], only 38.2% of the compounds fell within the respected range for the BIP*_caco-2_* criterion. Histograms showing the predicted qualitative human oral absorption parameter (in the scale 1 = low, 2 = medium and 3 = high) are represented in Figure S2. It was observed that 55.8% of the compounds in AfroDb were predicted to have high human oral absorption. The predicted % human oral absorption (on 0 to 100% scale) shows a similar trend, 47.0% of the compounds being predicted to be absorbed at 100% and 63.1% of the compounds predicted to be absorbed at >90%.

The size of a molecule, as well as its capacity to make hydrogen bonds, its overall lipophilicity and its shape and flexibility are important properties to consider when determining permeability. Molecular flexibility has been seen as a parameter which is dependent on the NRB, a property which influences bioavailability in rats [36]. As previously mentioned, the distribution of the NRB for this dataset showed a peak value lying between 3 and 4 RBs, with an average value of 6.3 (Table 2). The gap between the average and peak values could be explained by some degree of conformational flexibility in some of the bulkiest NPs (having as many as 61 RBs).

### Prediction of Blood–brain Barrier (BBB) Penetration

**Figure 7:**
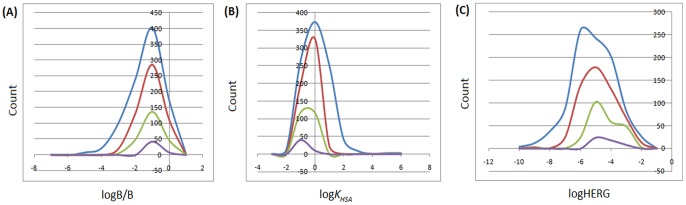
Distibution curves for some computed ADME parameters. (A) logB/B, (B) log*K*_HSA_, (C) logHERG. For subfigure B, the *x*-axis label is the lower limit of binned data, e.g. −2 is equivalent to −2 to −1. The colour codes are according to Figure 5.

The BBB partition coefficient is a good indicator of the ability of a drug to have access to the central nervous system (CNS). Drugs which are too polar do not cross the BBB. The blood/brain partition coefficients (logB/B) were computed and used as a predictor for access to the CNS. The predicted CNS activity was computed on a −2 (inactive) to +2 (active) scale and showed that only 3.8% of the compounds in AfroDb could be active in the CNS (predicted CNS activity >1). A distribution of logB/B (Figure 7A) showed a Gaussian-shaped curve with a peak at −0.5 logB/B units (the same for all the standard subsets), with >96% of the compounds in AfroDb falling within the recommended range for the predicted brain/blood partition coefficient (−3.0 to 1.2). Madin-Darby canine kidney (MDCK) monolayers, are widely used to make oral absorption estimates, the reason being that these cells also express transporter proteins, but only express very low levels of metabolizing enzymes [36]. They are also used as an additional criterion to predict BBB penetration. Thus, our calculated apparent MDCK cell permeability could be considered to be a good mimic for the BBB penetration (for non-active transport). It was estimated that about 53% of the compounds had apparent MDCK cell permeability values falling within the recommended range of 25–500 nm s^−1^ for 95% of known drugs. This situation was not improved in the “drug-like” and “lead-like” subsets (∼55% and ∼44% respectively).

### Prediction of Dermal Penetration

This factor is important for drugs administered through the skin. The distribution of computed skin permeability parameter, 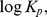 showed smooth Gaussian-shaped graphs centred at −2.5 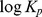 units for all 4 datasets (Figure S3), with ∼92% of the compounds in the AfroDb database falling within the recommended range for >95% of known drugs. The predicted maximum transdermal transport rates, 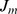 (in µ cm^−2^ hr^−1^), were computed from the predicted aqueous solubility (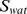) and skin permeability (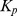), using the relation (2):
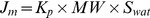
(2)


This parameter showed variations from 0 to >1,600 µ cm^−2^ hr^−1^, with only about 2.5% of the compounds in AfroDb having predicted value of 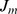 >100 µ cm^−2^ hr^−1^.

### Prediction of Plasma-protein Binding

The degree to which a drug binds to the blood plasma proteins may seriously affect its efficacy. This is because binding of drugs to plasma proteins (like human serum albumin, lipoprotein, glycoprotein, α, β, and γ globulins) greatly reduces the quantity of the drug in general blood circulation and hence the less bound a drug is, the more efficiently it can traverse cell membranes or diffuse. The predicted plasma-protein binding has been estimated by the prediction of binding to human serum albumin; the 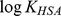 parameter (recommended range is −1.5 to 1.5 for 95% of known drugs). Figure 7B shows the variation of this calculated parameter within the AfroDb dataset, as well as for the standard subsets. This equally gave smooth Gaussian-shaped curves centred on 0.5 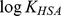 units for the total and “drug-like” libraries and −0.5 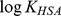 units for the “lead-like” and “fragment-like” subsets. In addition, our calculations revealed that >92% of the compounds within the AfroDb library complied with this parameter, which is an indication that a bulk of the compounds are likely to circulate freely within the blood stream and hence have access to their respective target sites.

### Metabolism Prediction

An estimated number of possible metabolic reactions has also been predicted by QikProp and used to determine whether the molecules can easily gain access to the target site after entering the blood stream. The average estimated number of possible metabolic reactions for the AfroDb library is between 6 and 7, while those of the standard subsets are respectively between 4 and 5, between 3 and 4 and between 2 and 3 (Table 2). Even though some of the compounds are likely to undergo as many as up to 25 metabolic reactions due to the complexity of some of the plant secondary metabolites within the database (Figure S4), up to about 80% of the compounds are predicted to undergo the recommended number of metabolic steps (1 to 8 reactions), with the situation improving to ∼91% and ∼97% in the “drug-like” and “lead-like” subsets respectively.

### Prediction of Blockage of Human Ether-a-go-go-related Gene Potassium (HERG K^+^) Channel

Human ether-a-go-go related gene (HERG) encodes a potassium ion (K^+^) channel that is implicated in the fatal arrhythmia known as *torsade de pointes* or the long QT syndrome [37]. The HERG K^+^ channel, which is best known for its contribution to the electrical activity of the heart that coordinates the heart’s beating, appears to be the molecular target responsible for the cardiac toxicity of a wide range of therapeutic drugs [38]. HERG has also been associated with modulating the functions of some cells of the nervous system and with establishing and maintaining cancer-like features in leukemic cells [39]. Thus, HERG K^+^ channel blockers are potentially toxic and the predicted IC_50_ values often provide reasonable predictions for cardiac toxicity of drugs in the early stages of drug discovery [40]. In this work, the estimated or predicted IC_50_ values for blockage of this channel have been used to model the process *in silico*. The recommended range for predicted log IC_50_ values for blockage of HERG K^+^ channels (logHERG) is>−5. A distribution curve for the variation of the predicted logHERG is shown in Figure 7C, which is a left-slanted Gaussian-shaped curve, with a peak at −5.5 logHERG units for the total library and −4.5 logHERG units for all the standard subsets. It was observed that, in general, this parameter was predicted to fall within the recommended range for about 58% of the compounds within the AfroDb database, ∼70% for the “drug-like” subset and ∼90% for the “lead-like” subset.

### Diversity Analysis

**Figure 8:**
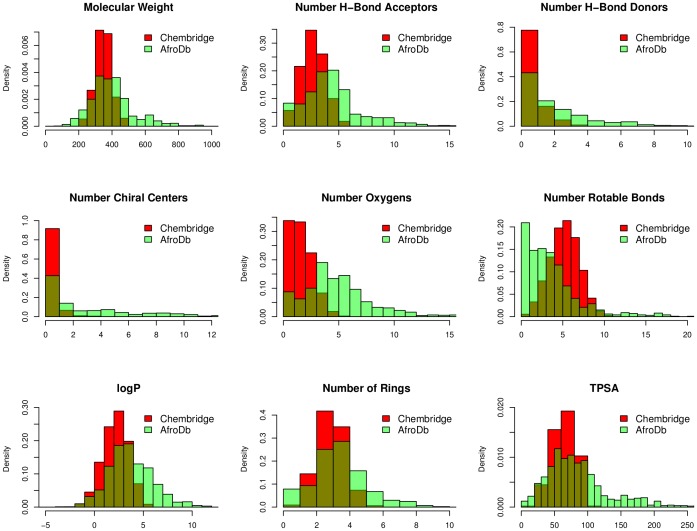
A simple descriptor-based comparison of the AfroDb database and the ChemBridge Diversity database. Comparison of typical physico-chemical property distributions (MW, HBA, HBD, NCC, NO, NRB, log *P*, NR and TPSA) in the AfroDb (green) and ChemBridge Diverset (red) database. All histograms and scatterplots were generated with the R software [85].

**Figure 9:**
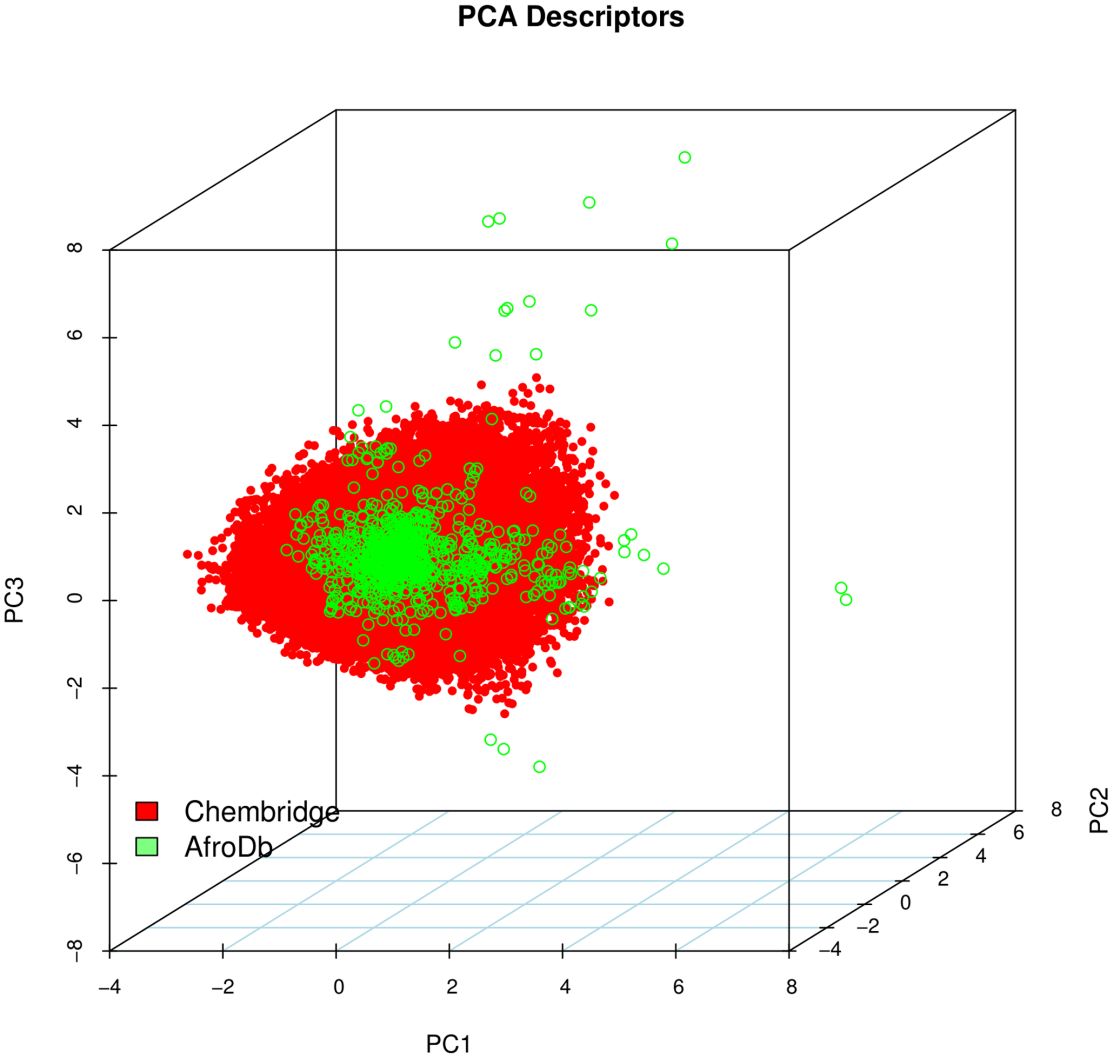
A principal component analysis (PCA) plot, showing the comparison of the chemical space defined by the NPs in AfroDb (green) and the chemical space represented by NPs in the ChemBridge Diversity (red) databases.

In order to reduce redundancy and enhance the coverage of biological activity and chemical space, a dataset for virtual screening must have the requirement of diversity. In this case, we carried out a simple molecular descriptor comparison with a relatively larger known diverse library (the DIVERSet™ Database, containing 48,651 compounds) from the ChemBridge Corporation [25]. Histograms showing the calculated descriptors; MW, HBA, HBD, log *P*, NRB,number of rings (NR), number of oxygens (NO), and total polar surface area (TPSA) are shown in Figure 8 for AfroDb (in light green) and the ChemBridge dataset (in red). The regions shown in dark green represent regions of intersection. The MW of the AfroDb dataset stretches up to about 1000 Da, while that of the ChemBridge dataset is restricted to the range 200≤ MW ≤500 Da. This observation could be explained by the complexity and large sizes of some of the compounds within the natural product library. The large proportion of very large and complex NPs in AfroDb, could also explain the average molar weight (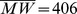
Da), when compared to those of the standard “drug-like”, “lead-like” libraries and typical drugs (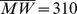
Da for typical drugs) [41]. This same explanation holds for the trend which is observed in the distributions of log *P*, HBD, NCC, NO, NRB, NR, TPSA and HBA for AfroDb, when compared with the ChemBridge dataset. It was generally observed that the AfroDb dataset covers a different physico-chemical space from the ChemBridge Diversity dataset. Principal component analysis (PCA) was as well used as a means of comparing the extent of diversity of the two datasets. This consists in reducing the dimensionality of the calculated descriptors by linearly transforming the data, by calculating a new and smaller set of descriptors, which are uncorrelated and normalised (mean = 0, variance = 1). The PCA scatter plot of the previously calculated physico-chemical properties of the AfroDb (light green) and ChemBridge Diverset database (red), shown in Figure 9, is a visual representation of the molecules in the respective datasets, as described by the 3 selected principal components (PC1, PC2 and PC3). Each point shown corresponds to a molecule, the spread of the points representing the diversity of the respective datasets. The first three principal components (PCs) explain 80.1% (AfroDb) and 63.7% (ChemBridge) of the variance of the individual datasets. The larger number of outliers in the case of the AfroDb dataset (away for the centre and towards the sides of the cube) indicates a wider sampling of the chemical space compared to the ChemBridge Diverset collection.

### Searching for Most Common Substructures

**Figure 10:**
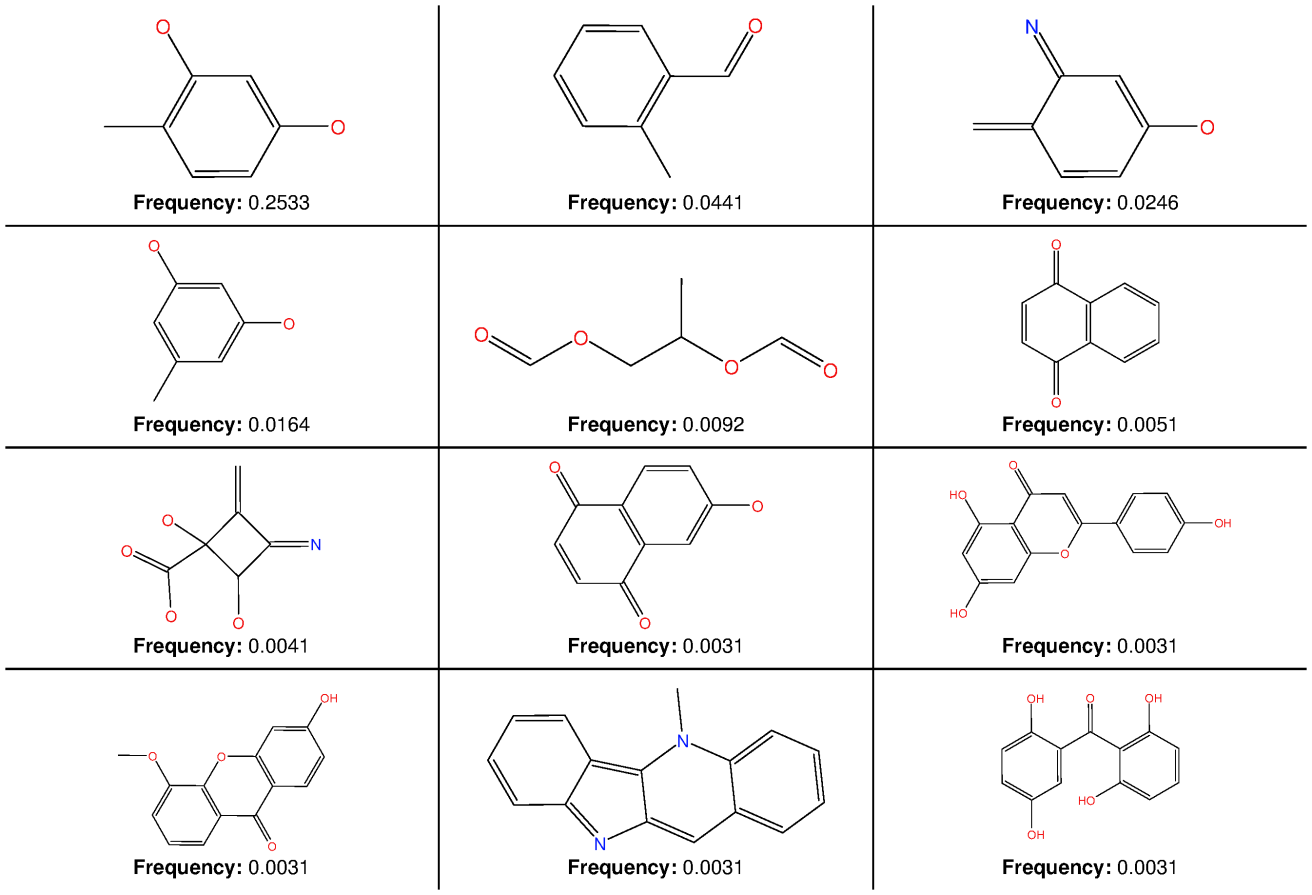
MCSS panel in AfroDb, featuring the most common cyclic structures included in the database.

The most common substructure selection (MCSS) panel for compound selection (Figure 10) is based on substructures that can be synthetically combined and are common in “drug-like” molecules and allows a direct selection and identification of compounds containing such substructures. The panel highlights the large diversity of the rings present in the NPs of AfroDb.

### Tautomer Generation and Synthetic Accessibility Evaluation

Of the 1008 compounds within AfroDb, we were able to identify 2308 possible tautomers, and respectively 1463, 524 and 103 possible tautomers for the standard subsets (“drug-like”, “lead-like” and “fragment-like”). The number of possible analogues were estimated at 9098, even the synthetic accessibility scores were quite weak for most of the proposed, as was expected for NPs.

### Some Selected Highly Potent Compounds

**Figure 11:**
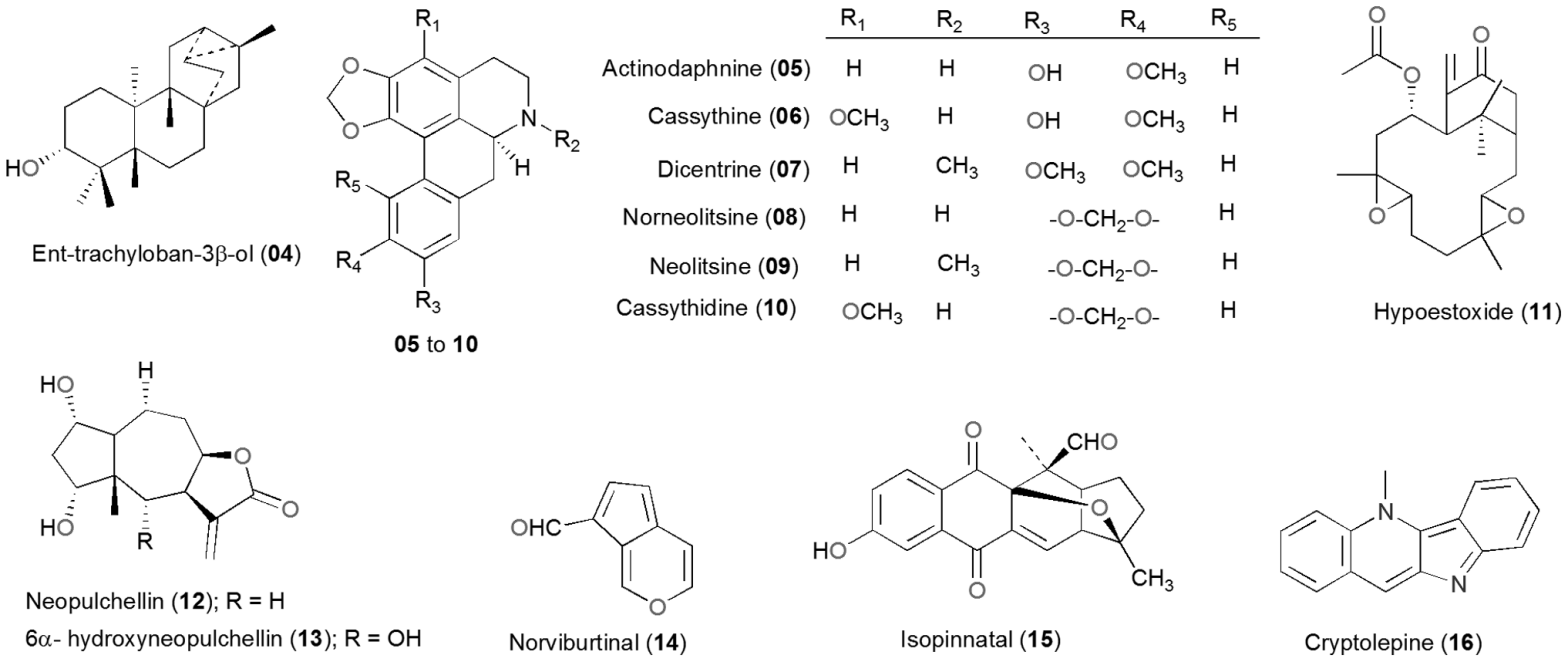
2D structures of selected promising compounds derived from the African flora and included in AfroDb.

Our discussion also includes selected compounds within the AfroDb database with promising biological activities, which have been isolated from African medicinal plants from the very first time. These have been summarised in Table 3, while their 2D structures are shown in Figure 11.

**Table 3:** Summary of selected promising potent compounds derived from African medicinal plants and currently included in AfroDb.

Compound	Plant source (country)	Measured activity(ies)	Reference
**04**	*Croton zambesicus* (Benin)	Induction of apoptosis in Human promyelocytic leukemia cells	[42]
**05–10**	*Cassytha filiformis* (Benin)	Cytotoxic, antitrypanosomal	[43]
**11**	*Hypoestoes rosea* (Nigeria)	Anti-inflammatory, antiangiogenic and antitumor activities,inhibiting the activity of IκB kinase	[44]–[46]
**12–13**	*Gaillardia aristata* (Egypt)	Anticancer	[47]
**14–15**	*Kigelia pinnata* (Zimbabwe)	Anticancer	[48]
**16**	*Sida acuta* (Cote d’Ivoire)	Anti-malarial	[56]

Among the promising compounds with anticancer activities, the trachylobane diterpene ent-trachyloban-3β-ol (**04**), derived from *Croton zambesicus* (Euphorbiaceae) in Benin, has been shown to exert a dose dependent cytotoxic effect, which varies between cell lines. Induction of apoptosis in HL-60 cells could be detected at a concentration of 50 µM after 24-h treatment. This compound was also able to induce apoptosis in human promyelocytic leukemia cells via caspase-3 activation in a concentration-dependent manner [42]. Other promising cytotoxic agents included are six aporphines (**05**–**10**) from *Cassytha filiformis*, among which actinodaphnine (**05**), cassythine (**06**), and dicentrine (**07**) were also shown to possess antitrypanosomal properties *in vitro* on *Trypanosoma brucei brucei* [43]. Hypoestoxide (HE, **11**), a natural diterpenoid [a bicyclo (9, 3, 1) pentadecane], derived from *Hypoestoes rosea* growing in Nigeria [44] has been reported to be a potent nonsteroidal anti-inflammatory drug. This compound is also reported to be non mutagenic and possesses antiangiogenic and antitumor activities, also inhibiting the activity of IκB kinase [45], [46]. Other potent anticancer compounds have been isolated from *Gaillardia aristata* growing in Egypt [47]. These include neopulchellin (**12**) and 6α- hydroxyneopulchellin (**13**), with respective IC_50_ values of 0.43 and 0.32 mgmL^-1^ against human cancer cell lines (breast (MCF7)) and 0.46, 0.34 mgmL^−1^ against human colon (HCT116), respectively. Examples of NPs with anticancer properties derived from plants growing in the Southern African region include norviburtinal (**14**) and isopinnatal (**15**). These compounds were derived from *Kigelia pinnata* harvested in Zimbabwe and have shown cytotoxic activity against various cancer cell lines after 144 h exposure [48].

The anti-malarial properties of some of the compounds included in the AfroDb dataset have been discussed in several review papers [49]–[53], as well as compounds with antiparasitic properties [54] and antimicrobial activities [55]. One of the promising anti-malarial compounds include cryptolepine (**16**) derived from *Sida acuta* (Malvaceae), a plant growing in Côte d’Ivoire, which traditional healers commonly use for the treatment of malaria [56]. This compound has shown antiplasmodial activity against the FcM29-Cameroon (chloroquine-resistant strain) and a Nigerian chloroquine-sensitive strain, with IC_50_ values of 0.13 and 0.17 µg/mL respectively.

### Usefulness of the AfroDb Library

It is important to mention that virtual screening results sometimes provide insight and direct natural product chemists to search for theoretically active principles with attractive ADMET profiles, which have been previously isolated, but not tested for activity against specified drug targets (if samples are absent). This “resurrection” process could prove to be a better procedure for lead search than the random screening, which is common practice in most laboratories in Africa. AfroDb is constantly being updated; meanwhile a MySQL platform to facilitate the searching of this database and ordering of compound samples is under development within our group and will also be published subsequently. However, 3D structures of the compounds, as well as their physico-chemical properties that were used to evaluate “drug-likeness” and the DMPK profile, can be freely downloaded in the (Dataset S1). In addition, information about compound sample availability can be obtained on request from the authors of this paper or from the pan-African Natural Products Library (p-ANAPL) project [57]–[58].

## Materials and Methods

### Data Sources and Cutoff Points for Biological Activities

The plant sources, geographical collection sites, chemical structures of pure compounds as well as their biological activities, were retrieved from literature sources comprising mainly published articles from across the major journals of natural product chemistry, as well as MSc and PhD theses, textbook chapters, as well as unpublished conference presentations (from personal communication with the authors). References were recorded from 1971 to 2013. Following criteria used by Mahmoudi et al. [59] and Wilcox et al. [60], a pure compound was considered highly active if IC_50_<0.06 µM, being active with 0.06 µM≤IC_50_≤5 µM, weakly active when 5 µM≤IC_50_≤10 µM and compounds with IC_50_>10 µM were considered inactive. Up to weakly active compounds were selected.

### Generation of 3D Models, Optimization and Calculation of Molecular Descriptors

Based on the known chemical structures of the NPs, all 3D molecular structures were generated using the graphical user interface (GUI) of the MOE software [61] running on a Linux workstation with a 3.5 GHz Intel Core2 Duo processor. The 3D structures were generated using the builder module of MOE and energy minimization was subsequently carried out using the MMFF94 force field [62] until a gradient of 0.01 kcal/mol was reached. The 3D structures of the compounds were then saved as.mol2 files subsequently included into a MOE database (.mdb) file and converted to other file formats (.sdf,.mol,.mol2 and.ldb), which are suitable for use in several virtual screening workflow protocols. Up to 10 possible tautomers were generated per compound in the dataset. The MW, NRB, log *P*, HBA, HBD and Lipinski violations were calculated using the molecular descriptor calculator included in the QuSAR module of the MOE package [61].

### Determination of ADMET Profiles

The previously prepared 1,008 low energy 3D chemical structures in the AfroDb library were saved in.mol2 format and initially treated with LigPrep [63], distributed by Schrodinger Inc. This implementation was carried out with the graphical user interface (GUI) of the Maestro software package [64], using the OPLS forcefield [65]–[67]. Protonation states at biologically relevant pH were correctly assigned (group I metals in simple salts were disconnected, strong acids were deprotonated, strong bases protonated, while topological duplicates and explicit hydrogens were added). A set of ADMET-related properties (a total of 46 molecular descriptors) were calculated by using the QikProp program [68] running in normal mode. QikProp generates physically relevant descriptors, and uses them to perform ADMET predictions. An overall ADME-compliance score – drug-likeness parameter (indicated by #stars), was used to assess the pharmacokinetic profiles of the compounds within the AfroDb library. The #stars parameter indicates the number of property descriptors computed by QikProp that fall outside the optimum range of values for 95% of known drugs. The methods implemented were developed by Jorgensen *et al*. [69]–[70] and among the calculated descriptors are: the total solvent-accessible molecular surface, 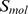 in Å^2^ (probe radius 1.4 Å) (range for 95% of drugs: 300–1000 Å^2^); the hydrophobic portion of the solvent-accessible molecular surface, 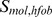 in Å^2^ (probe radius 1.4 Å) (range for 95% of drugs: 0–750 Å^2^); the total volume of molecule enclosed by solvent-accessible molecular surface, 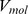 in Å^3^ (probe radius 1.4 Å) (range for 95% of drugs: 500–2000 Å^3^); the logarithm of aqueous solubility, 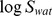 (range for 95% of drugs: −6.0 to 0.5) [69], [71]; the logarithm of predicted binding constant to human serum albumin, 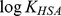 (range for 95% of drugs: −1.5 to 1.2) [72]; the logarithm of predicted blood/brain barrier partition coefficient, logB/B (range for 95% of drugs: −3.0 to 1.0) [73]–[75]; the predicted apparent Caco-2 cell membrane permeability (BIP*_caco-2_*) in Boehringer–Ingelheim scale, in nm/s (range for 95% of drugs: <5 low, >100 high) [76]–[78]; the predicted apparent Madin-Darby canine kidney (MDCK) cell permeability in nm s^−1^ (<25 poor, >500 great) [77]; the index of cohesion interaction in solids, Ind_coh_, calculated from the HBA, HBD and the surface area accessible to the solvent, SASA (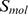) by the relation Ind_coh_ = HBA
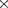
HBD^1/2^/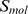 (0.0 to 0.05 for 95% of drugs) [71]; the globularity descriptor, Glob = 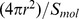, where *r* is the radius of the sphere whose volume is equal to the molecular volume (0.75 to 0.95 for 95% of drugs); the predicted polarizability, 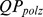 (13.0 to 70.0 for 95% of drugs); the predicted logarithm of IC_50_ value for blockage of HERG K^+^ channels, logHERG (concern<−5) [79]–[80]; the predicted skin permeability, 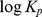 (−8.0 to −1.0 for 95% of drugs) [81]–[82]; and the number of likely metabolic reactions, #metab (range for 95% of drugs: 1–8).

### Diversity Analysis and Searching with Most Common Substructures

The ChemBridge Diverset dataset (48,651 compounds) was downloaded from the official ChemBridge webpage [25]. The MW, NRB, log *P*, log *S*, HBA, HBD, THSA, TPSA, NO, NCC, NR and number of Lipinski violations were calculated using the molecular descriptor calculator included in the QuSAR module of the MOE package [61]. The LibMCS program of JKlustor [83] was used for maximum common substructure clustering of the AfroDb database. In the MCSS search, only structures with MW ≤700 were included, since MCSS clustering is only feasible on small molecules. This means, only 975 of the 1008 compounds of the AfroDb were analyzed for MCSS. The compounds were fragmented using the RECAP algorithm [84] and the frequency of the generated fragments were analysed, leading to the identification of the most frequent substructures.

## Conclusions

To the best of our knowledge, AfroDb represents the largest “drug-like” and diverse collection of 3D structures of NPs covering the geographical region of the entire African continent, which is readily available for download and use in virtual screening campaigns. Virtual screening workflows usually involve docking a compound library into the binding site of a target receptor and using scoring functions and binding free energy calculations to identify putative binders. The availability of 3D structures of the compounds to be used for docking is of utmost importance. Therefore the availability of such structures within AfroDb, as well as their calculated physico-chemical properties and indicators of “drug-likeness” within this newly developed database will facilitate the drug discovery process from leads that have been identified from African medicinal plants.

## Supporting Information

Figure S1
**Distribution curves for compliance to Jorgensen’s “Rule of Three”.** (A) calculated 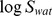 against count, (B) predicted BIP*_caco-2_* against count. Colour codes are as defined in Figure 6.(TIF)

Figure S2 **Histograms showing the distribution of human oral absorption predictions.**
(TIF)

Figure S3 **Distribution curves for the predicted skin penetration parameter.** Colour codes are as defined in Figure 6.
(TIF)

Figure S4 **Graphs showing the distribution of the predicted number of metabolic reactions for compounds in AfroDb.** Colour codes are as defined in Figure 6.
(TIF)

Dataset S1 **SDF 3D structures of compounds currently included in AfroDb, along with computed physico-chemical descriptors used to predict drug-likeness and DMPK profile.** This file is saved in.sdf format (which can be viewed using many drug discovery software including Maestro, MOE, Discovery studio, LigandScout, etc.) or into.ldb format using the software LigandScout. Requests for other file formats could be addressed to the authors of this article.
(SDF)
