# Supplementary figures and images for: AfroDb: A Select Highly Potent and Diverse Natural Product Library from African Medicinal Plants
Source: PLoS One. 2013 Oct 30;8(10):e78085. doi: 10.1371/journal.pone.0078085 (PMC3813505; doi:10.1371/journal.pone.0078085)

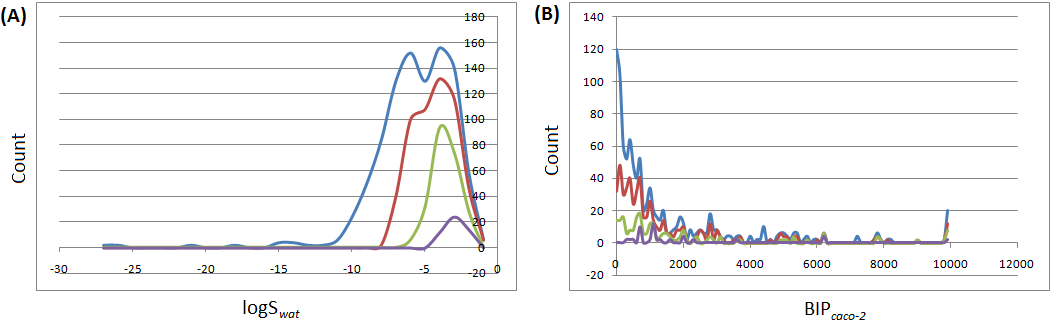

Supplement: Figure S1 — Distribution curves for compliance to Jorgensen’s “Rule of Three”. (A) calculated against count, (B) predicted BIPcaco-2 against count. Colour codes are as defined in Figure 6. (TIF) [file pone.0078085.s001.tif]

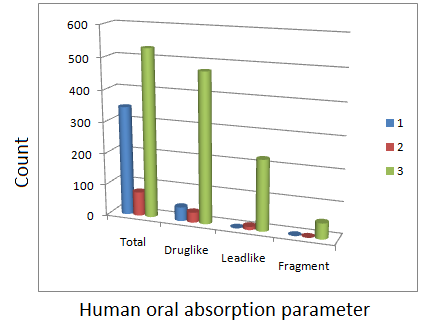

Supplement: Figure S2 — Histograms showing the distribution of human oral absorption predictions. (TIF) [file pone.0078085.s002.tif]

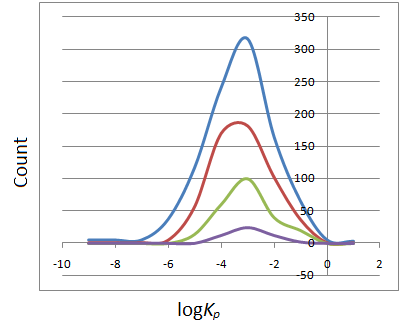

Supplement: Figure S3 — Distribution curves for the predicted skin penetration parameter. Colour codes are as defined in Figure 6. (TIF) [file pone.0078085.s003.tif]

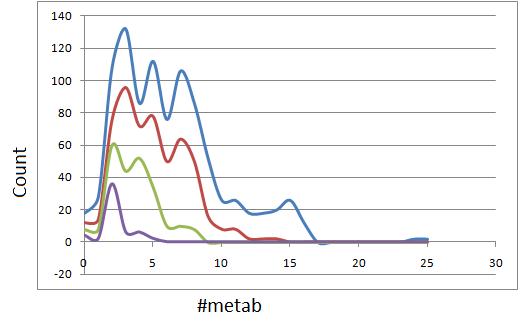

Supplement: Figure S4 — Graphs showing the distribution of the predicted number of metabolic reactions for compounds in AfroDb. Colour codes are as defined in Figure 6. (TIF) [file pone.0078085.s004.tif]
